# Host range, transmissibility and antigenicity of a pangolin coronavirus

**DOI:** 10.1038/s41564-023-01476-x

**Published:** 2023-09-25

**Authors:** Yixuan J. Hou, Shiho Chiba, Sarah R. Leist, Rita M. Meganck, David R. Martinez, Alexandra Schäfer, Nicholas J. Catanzaro, Vishwaraj Sontake, Ande West, Catlin E. Edwards, Boyd Yount, Rhianna E. Lee, Samuel C. Gallant, Seth J. Zost, John Powers, Lily Adams, Edgar F. Kong, Melissa Mattocks, Aleksandra Tata, Scott H. Randell, Purushothama R. Tata, Peter Halfmann, James E. Crowe, Yoshihiro Kawaoka, Ralph S. Baric

**Affiliations:** 1 Department of Epidemiology, University of North Carolina at Chapel Hill, Chapel Hill, NC USA; 2 Influenza Research Institute, Department of Pathobiological Sciences, School of Veterinary Medicine, University of Wisconsin, Madison, WI USA; 3 Department of Cell Biology, Regeneration Next Initiative, Duke University Medical Center, Durham, NC USA; 4 Marsico Lung Institute, University of North Carolina at Chapel Hill, Chapel Hill, NC USA; 5 Vanderbilt Vaccine Center, Vanderbilt University Medical Center, Nashville, TN USA; 6 Division of Virology, Department of Microbiology and Immunology, Institute of Medical Science, University of Tokyo, Tokyo, Japan; 7 Department of Microbiology and Immunology, University of North Carolina at Chapel Hill, Chapel Hill, NC USA; 8 Present Address: Moderna Inc., Cambridge, MA USA

**Keywords:** SARS-CoV-2, Viral transmission

## Abstract

The pathogenic and cross-species transmission potential of SARS-CoV-2-related coronaviruses (CoVs) remain poorly characterized. Here we recovered a wild-type pangolin (Pg) CoV GD strain including derivatives encoding reporter genes using reverse genetics. In primary human cells, PgCoV replicated efficiently but with reduced fitness and showed less efficient transmission via airborne route compared with SARS-CoV-2 in hamsters. PgCoV was potently inhibited by US Food and Drug Administration approved drugs, and neutralized by COVID-19 patient sera and SARS-CoV-2 therapeutic antibodies in vitro. A pan-*Sarbecovirus* antibody and SARS-CoV-2 S2P recombinant protein vaccine protected BALB/c mice from PgCoV infection. In K18-hACE2 mice, PgCoV infection caused severe clinical disease, but mice were protected by a SARS-CoV-2 human antibody. Efficient PgCoV replication in primary human cells and hACE2 mice, coupled with a capacity for airborne spread, highlights an emergence potential. However, low competitive fitness, pre-immune humans and the benefit of COVID-19 countermeasures should impede its ability to spread globally in human populations.

## Abstract (web summary)

Pangolin coronavirus GD has reduced replication and transmission fitness compared with SARS-CoV-2, and therapies approved for SARS-CoV-2 are effective against this virus.

## Main

The coronavirus disease 2019 (COVID-19) pandemic is caused by severe acute respiratory disease syndrome coronavirus 2 (SARS-CoV-2), which emerged in southern China, causing >750 million infections and >6.9 million deaths. SARS-CoV-2 most probably emerged from animal reservoirs in Southeast Asia and is efficiently transmitted between humans^1,2^. Although an intermediate species remains elusive, Malayan pangolins (*Manis javanica*) represent one of several potential species^3–6^. Pangolins harbour diverse Sarbecoviruses that are genetically related to SARS-CoV-2, as well as strains circulating in horseshoe bats (*Rhinolophus*)^3,4,7–11^. While none of these strains are considered the direct progenitor of SARS-CoV-2, the pangolin (Pg) CoV strain GD, bat CoVs BANAL-52 and BANAL-103 strains encode receptor binding domains (RBD) that display remarkable homology with the SARS-CoV-2 RBD^3,11^. These closely related SARS-2-like CoVs lack a polybasic furin cleavage site (RRAR) at the S1/S2 junction of the SARS-CoV-2 spike protein, which is thought to promote SARS-CoV-2 spike RBD-ACE2 receptor binding^12^, pathogenesis^13^, transmission^14^ and emergence^15^. However, it is unclear whether these related strains can efficiently infect and replicate in primary human cells, undergo airborne transmission between hosts and cause severe disease in mammals.

Micro-variation in the SARS-CoV-2 RBD alters species-specific receptor usage and transmission potential in mammals, and humans transmit virus to other mammalian species such as mink, feline and Cervidae populations^16–18^. The discovery of SARS-2-like bat CoV BANAL-52 and PgCoV GD support previous hypotheses that many zoonotic CoVs are pre-determined to replicate efficiently in multiple mammalian hosts, including humans^19,20^. The earliest known PgCoV strains, including the GD and GX clusters, were identified in 2 batches of smuggled pangolins. For the GD strain, the same metagenomic data from a single source sample were reported in different publications^3,6,21^, triggering confusion about origin and authenticity. While human infections have not been reported, pseudotyped viruses encoding the PgCoV GD spike can utilize human ACE2 receptor for entry^9^. However, PgCoV replication and transmission efficiency, tropism and fitness in primary human respiratory epithelial cells remain uncertain^21^, as does its ability to replicate and transmit in small animal models. Lastly, the performance of current COVID-19 antiviral drugs, vaccines and therapeutic antibodies to protect from live virus PgCoV zoonotic events in vivo is important for global health preparedness.

## Results

### Recovery and characterization of PgCoV GD strain in vitro

**Fig. 1:**
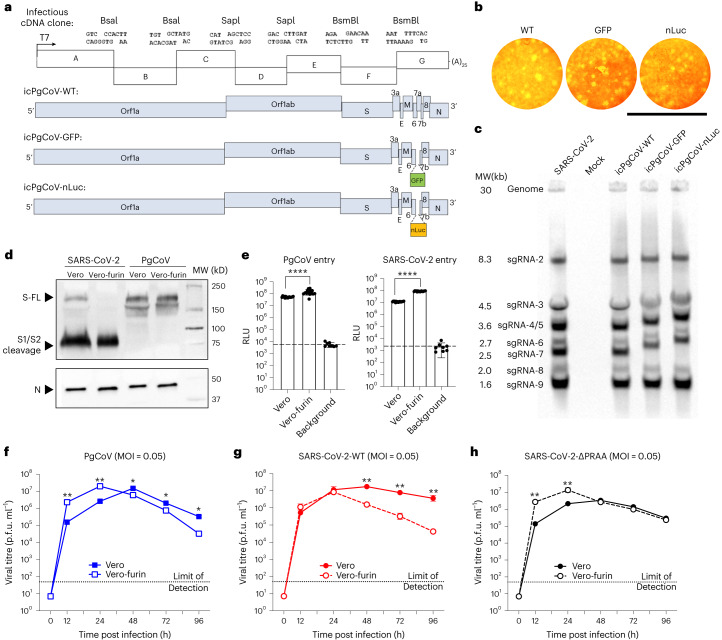
Generation and characterization of recombinant PgCoVs. **a**, Schematic design of PgCoV GD1 infectious cDNA clone. **b**, Plaque formation of the three recombinant PgCoVs. Scale bar, 8 mm. **c**, Northern blot analysis of genomic and subgenomic mRNAs isolated from SARS-CoV-2 and PgCoV infected cells at 24 h. **d**, Western blot analysis of semi-purified PgCoV and SARS-CoV-2 virions cultured in Vero or Vero-furin cell lines identified as full-length (FL), S1/S2 cleaved spike protein (S) and nucleocapsid protein (N). Samples were loaded on the basis of an equal amount of the N protein; this western blot was repeated twice with the same result. **e**, Efficient entry of PgCoV-nLuc and SARS-CoV-2-nLuc recombinant viruses into Vero-81 and Vero-furin cells at an MOI of 2. After 1 h infection, viruses were removed and cells were treated with neutralization antibodies to minimize secondary rounds of infection. The RLU representing the nLuc expression level was measured at 12 h post infection (*n* = 8 replicates per group, data are mean ± s.d.) and analysed using unpaired *t*-test; ****P < 0.0001. **f**–**h**, Multistep growth curves of PgCoV-WT (**f**), SARS-CoV-2-WT (**g**) and SARS-CoV-2-∆PRRA (**h**) in Vero and Vero-furin cells. Source data

The PgCoV GD genome shares 90.1% identity with SARS-CoV-2 and encodes 90.7% and 96.7% of homologous residues in the full-length spike protein and RBD, respectively (Supplementary Fig. 1a,b). To evaluate whether the PgCoV genome is viable in cell culture, a synthetic complementary (c)DNA clone was generated from the full-length PgCoV GD strain sequence^22–25^, including derivatives that replaced ORF7a with either the green fluorescent protein (GFP) or the nano-luciferase (nLuc) reporter genes. After assembly of the multicomponent cDNA clone, full-length viral RNA genomes were transcribed in vitro using a T7-RNA polymerase and then electroporated into Vero-E6 cells. Cytopathic effects (CPEs) were extensive by day 3–4 post infection and recombinant viruses formed visible plaques (Fig. 1a,b). To evaluate PgCoV GD transcription, northern blot analysis demonstrated full length and eight subgenomic mRNA (sgRNA), phenocopying SARS-CoV-2 transcription profiles (Fig. 1c). As expected, the molecular weights of sgRNA2 to sgRNA7 were visibly increased in PgCoV derivatives encoding the larger GFP and nLuc reporter transgenes as compared with the wild-type (WT) virus.

Group 1b Sarbecoviruses may encode a furin cleavage site at the S1/S2 boundary and/or a less efficient site in S2 (refs. ^26–28^). To evaluate the expression and processing of the PgCoV spike glycoprotein which lacks a polybasic cleavage site at the S1/S2 boundary, western blot analysis of PgCoV-WT and SARS-CoV-2 WT (strain WA1) virions were compared in infected wild-type Vero-E6 or in a previously described furin-overexpressing Vero-E6 cell line (Fig. 1d)^29^. SARS-CoV-2 grown in wild-type Vero cells displayed both full-length and S1/S2 processed spike glycoproteins, whereas the S1/S2 cleavage products were prominent in virions acquired from Vero-furin cells. In contrast, the PgCoV glycoprotein spike was insensitive to S1/S2 cleavage, even in furin-overexpressing cell lines, although the presence of a ~140–160 kDa spike product in PgCoV infected cells may reflect limited cleavage at a second S2 furin cleavage site, reported in SARS-CoV and PgCoV strains^27^. Next, we compared PgCoV-nLuc and SARS-CoV-2-nLuc entry efficiency in Vero and Vero-furin cells, the latter expressing high levels of furin (Fig. 1e)^29^. Both viruses display enhanced entry in the Vero-furin cells compared with Vero-E6 cells, as evidenced by ~5- and ~10-fold significantly increased levels of nLuc expression in PgCoV and SARS-CoV-2 infected cultures, respectively, through 48 h.

To compare virus growth, we performed a multistep growth curve analysis in both Vero cell types (Fig. 1f,g). To investigate the role of the furin cleavage site in SARS-CoV-2 entry, we generated a recombinant SARS-CoV-2 mutant ∆PRRA lacking the S1/D2 furin cleavage site (Supplementary Fig. 1c) and compared the growth kinetics of PgCoV GD, SARS-CoV-2 and SARS-CoV-2 ∆PRRA in culture (Fig. 1h). In Vero-furin cells, all three viruses replicated to similar titres of ~7 × 10^7^ plaque forming unit (p.f.u.) ml^−1^ at 24 h. At early times, SARS-CoV-2 replicated efficiently in both cell lines, although virus titre decay rates were accelerated in Vero-furin cell lines. Importantly, PgCoV GD growth characteristics most closely approximated the phenotypes seen with the furin-site knockout virus SARS-2-∆PRRA in both cell lines. Together, these data support earlier studies that showed that the S1/S2 furin cleavage facilitates SARS-CoV-2 entry and replication efficiency in vitro^13^, but that other factors may also contribute.

### Mammalian ACE2 usage and sensitivity to antiviral drugs

**Fig. 2:**
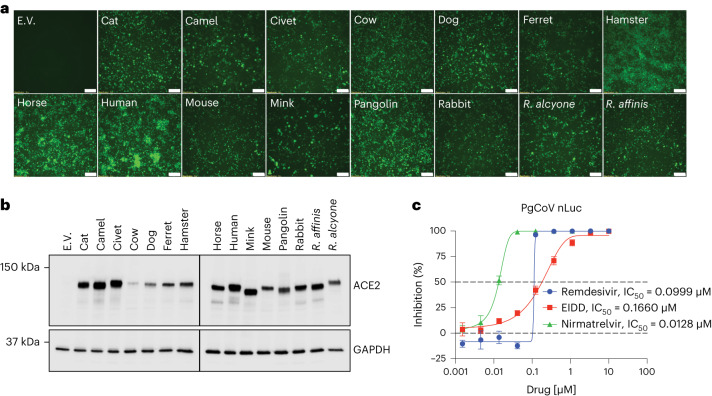
PgCoV GD strain displays a host range similar to that of SARS-CoV-2. **a**, GFP signals were observed in DBT9 cells expressing ACE2 orthologues or empty vector (E.V.) infected with icPgCoV-GFP at 24 h post infection. Scale bar, 100 μM. **b**, Western blot analysis of ACE2 orthologue or E.V. expression from 15 mammalian species in DBT9 cells. **a** and **b** were repeated twice with the same results. **c**, In vitro potency of RDV, EIDD and nirmatrelvir against icPgCoV in A549-hACE2 cells based on RLU of icPgCoV replication in the presence of decreasing concentrations of RDV, EIDD and nirmatrelvir in A549-hACE2 cells. Quantitation of icPgCoV-nLuc replication as measured by nLuc in technical triplicates. Shown are the IC_50_ values of RDV, EIDD and nirmatrelvir, *n* = 3 replicates per group, data are mean ± s.d. Source data

The PgCoV GD S RBD is highly homologous with SARS-CoV-2 (Supplementary Fig. 1d). To examine the receptor specificities of PgCoV GD, we used non-permissive cells stably transfected with ACE2 receptors derived from pangolin, human and many other mammalian species (Fig. 2). Using PgCoV GD GFP fluorescent viruses, we demonstrate robust virus replication in all the cell lines except non-permissive cells, although some differences in fluorescence were noted (Fig. 2a). Using western blot analyses with anti-ACE2 antibody, we further demonstrate high levels of mammalian ACE2 orthologue receptor expression from each species, but endogenous ACE2 expression was not detected in non-permissive cells (Fig. 2b).

Given the broad host range of PgCoV GD, we evaluated virus sensitivity to US Food and Drug Administration (FDA)-approved drugs such as remdesivir (RDV) and molnupiravir (EIDD), which have broad antiviral activity against many coronaviruses^30,31^. Using A549-hACE2 cells^32^, the PgCoV GD nLUC reporter virus was highly sensitive to current FDA-approved drugs such as RDV, EIDD and the oral protease inhibitor nirmatrelvir, demonstrating half-maximal inhibitory concentration (IC_50_) values of 0.099, 0.166 and 0.0128 μM, respectively (Fig. 2c). Together, these data not only demonstrate the broad host range potential of PgCoV GD but also revealed its high sensitivity to FDA-approved small-molecule inhibitors that target conserved nsp12 polymerase and nsp5 protease activities.

### PgCoV replicates efficiently in primary human airway cells

**Fig. 3:**
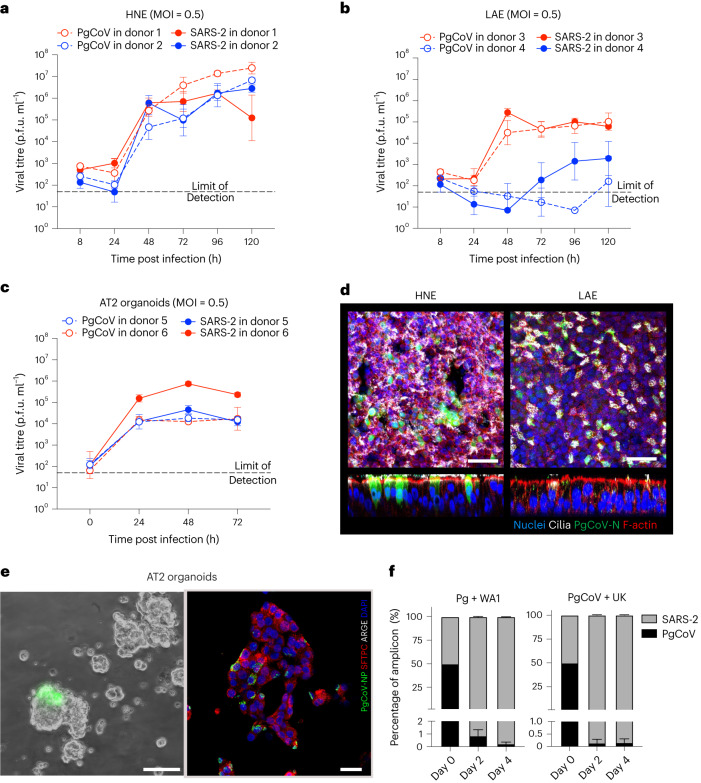
Susceptibility, adaptation and fitness of PgCoV in human respiratory cells. **a**–**c**, Comparison of PgCoV-WT and SARS-CoV-2 (WA1) growth in primary HNE cells (**a**), LAE cells (**b**) and AT2 organoids (**c**). The growth curve values were measured in triplicate for each timepoint, data are mean ± s.d. **d**, Immunofluorescence co-staining of PgCoV-WT antigens in representative HNE and LAE cultures at 72 h post infection. Scale bar, 50 μm. **e**, Live image of PgCoV-GFP virus-infected AT2 organoids at 48 h (left; scale bar, 40 μm) and immunofluorescence co-staining (right; scale bar, 25 μm) of PgCoV-WT (green) with SFTPC (red) and AGER (white) on infected AT2 organoids fixed at 72 h. **f**, Percentage of NTD coding sequences in RNA samples collected from PgCoV and SARS-CoV-2 competition experiments, *n* = 3 samples per timepoint, data are mean ± s.d. Source data

To evaluate the ability of PgCoV to replicate in human respiratory tissues, primary human cells from large airway epithelia (LAE) and nasal epithelia (HNE) from different donors (*n* = 2 per cell type) were infected with either PgCoV-WT or SARS-CoV-2 WA1 strain at an MOI of 0.5 (Fig. 3). Although donor variation influenced replication efficiency, PgCoV exhibited slightly increased or equivalent growth, as compared with SARS-CoV-2. In HNE, PgCoV replicated to titres of 6.6 × 10^6^ and 1.62 × 10^7^ p.f.u. ml^−1^ at 5 d post infection, whereas SARS-CoV-2 peaked at ~2-fold lower titres of 3.16 × 10^6^ and 6.6 × 10^6^ p.f.u. ml^−1^, respectively (Fig. 3a). In one LAE culture, PgCoV and SARS-CoV-2 titres plateaued between ~10^4^ and 10^5^ p.f.u. ml^−1^, while another patient code did not efficiently support the growth of either virus (Fig. 3b). Similar variation in *Sarbecovirus* growth across different patient codes has been reported previously^22^. Immunofluorescence staining of PgCoV antigens demonstrated that ciliated nasal and bronchial epithelial cells were the primary targets of viral infection (Fig. 3d), recapitulating SARS-CoV-2 tropism for ciliated cells in these tissues^22^.

SARS-CoV-2 primarily infects alveolar type II (AT2) pneumocytes, which maintain alveolar homoeostasis and gas exchange, and whose loss is correlated with severe disease outcomes during COVID-19 infection^22^. To evaluate the ability of PgCoV replication in distal lung tissues, human AT2 organoid cultures from two donors^33^ were infected with PgCoV-WT or SARS-CoV-2 at an MOI of 0.5 (Fig. 3c). In the AT2 organoids from donor 5, SARS-CoV-2 replicated to 7.41 × 10^5^ p.f.u. ml^−1^, a log higher than the peak PgCoV titre of 4.57 × 10^4^ p.f.u. ml^−1^. In organoid patient code 6, both viruses displayed similar growth, peaking at titres of ~10^4^ p.f.u. ml^−1^. The replication of PgCoV in AT2 organoids was also confirmed by immunofluorescence analysis (Fig. 3e), which demonstrated expression of viral antigens in close proximity with surfactant protein C (SFTPC)^33^. Together, these data indicate that PgCoV can replicate efficiently in human respiratory tissues from the proximal to the distal lung.

To compare replication fitness, competition assays were performed in LAE cultures simultaneously infected at an MOI of 1 each for PgCoV-WT and either SARS-CoV-2 strain WA1 or B.1.1.7 (UK variant). After 4 d, viral RNAs from progeny virions collected at 48 h intervals were quantified by deep sequencing of the variable spike N-terminal domain (NTD) (Fig. 3f). After 48 h, both WA1 and B.1.1.7 dominated the culture, with more than 98% of the RNA amplicons derived from these human strains. Together, these data suggest that the competitive fitness of PgCoV is lower than these early SARS-CoV-2 strains.

### Airborne transmission of PgCoV in hamsters

**Fig. 4:**
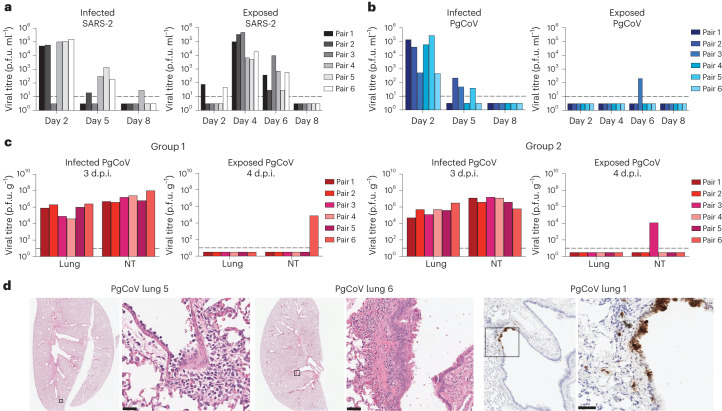
PgCoV transmits less efficiently than SARS-CoV-2 in hamster models. **a**–**c**, Viral titres in nasal washes were determined from infected and exposed hamster pairs in SARS-CoV-2 (**a**) and 3 replicate experiments involving PgCoV groups (**b** and **c** (groups 1 and 2)). Uninfected groups of exposed animals were housed in separate cages with a 5-cm distance from the infected hamster. d.p.i., days post infection. **d**, PgCoV disease pathology in the lung (left) and immunohistology staining for PgCoV N protein (right). Boxes on left identify tissue regions examined under higher magnification on right.

PgCoV efficiently uses the hamster ACE2 receptor for entry (Fig. 2), so hamsters were intranasally infected with 10^3^ p.f.u. of PgCoV or SARS-CoV-2. By 2–3 d, PgCoV replicated to titres of ~7.0 × 10^4^ p.f.u. g^−1^ and ~6.0 × 10^5^ p.f.u. g^−1^ in lung and nasal turbinate tissues, respectively, similar to titres achieved by SARS-CoV-2 (Fig. 4). At 4 d post infection, all hamsters developed mild broncho-interstitial pneumonia, with pathology scores ranging from 3 of 16 to 6 of 16, depending on the animal (Fig. 4d and Supplementary Table 1). Immunohistochemistry signals were prominent in bronchiolar and alveolar epithelial cells (pneumocytes) and presumed to be macrophages. To evaluate the airborne transmission potential, 18 or 6 hamsters were infected with PgCoV GD or SARS-CoV-2, as described in earlier studies^34–36^. After 1 d, each infected hamster was housed adjacent to an uninfected recipient hamster (Fig. 4a–c), separated by a wire-mesh divider (5 cm) to acquire airborne transmission. Viral titres in nasal wash demonstrated that SARS-CoV-2 and PgCoV GD replicated to similar titres on day 2, although PgCoV appeared to clear faster at later times. While SARS-CoV-2-infected donors rapidly transmitted virus to 100% of the uninfected recipients after 3–4 d (6/6), only three hamsters in the PgCoV donor group successfully transmitted the infectious virus to an uninfected recipient (3/18) by day 6, demonstrating a significantly lower airborne transmission rate (~16.6%), as compared with SARS-CoV-2 (*P* < 0.001, Fisher exact test) (Fig. 4a–c).

### PgCoV neutralization phenotypes

**Fig. 5:**
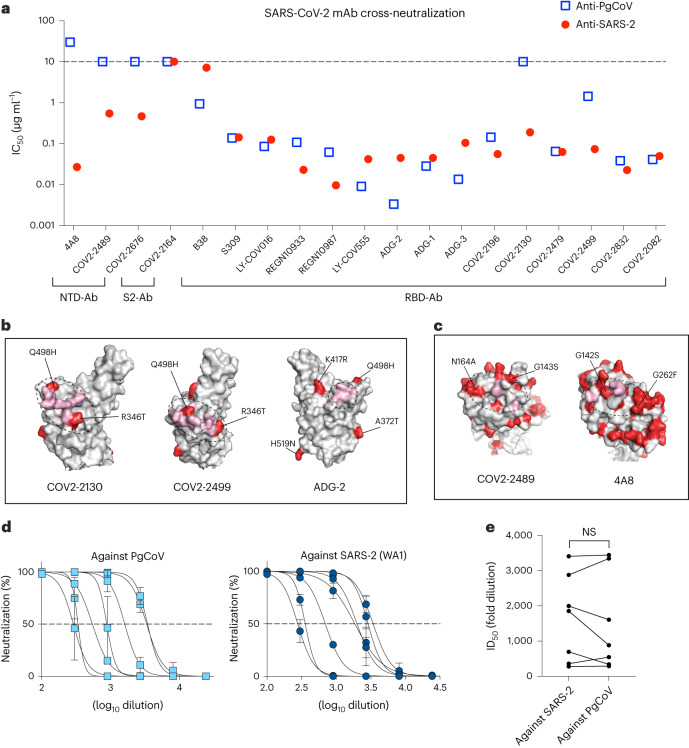
SARS-CoV-2-specific antibodies neutralized PgCoV infection in vitro and in vivo. Neutralization assays were performed using SARS-CoV-2 nLUC and PgCoV-nLUC recombinant viruses and panels of neutralizing monoclonal antibodies and convalescent patient sera. **a**, Summary of IC_50_ values from a panel of SARS-CoV-2 neutralizing antibodies. **b**, RBD antibody binding sites (pink) impacted by PgCoV natural variation (red). **c**, NTD antibody binding sites (pink) impacted by PgCoV natural variation (red). Binding sites in **b** and **c** were annotated on the basis of a SARS-CoV-2 spike structure (PBD ID: 6zp7). **d**, COVID-19 patient sera 8-point neutralization curves against SARS-CoV-2 (WA1) and PgCoV; each sample was run in triplicate in the assay, data are mean ± s.d. **e**, COVID-19 ID_50_ neutralizing titres against SARS-CoV-2 and PgCoV. Values were analysed using paired *t*-test; NS, *P* > 0.05.

The PgCoV GD spike protein has ~10% amino acid variation when compared with the prototype SARS-CoV-2 WA1 strain that encodes significant variation in spike NTD (Supplementary Fig. 1a). The PgCoV GD RBD also contains 6 amino acid changes (R346T, A372T, I402V, K417R, Q498H and H519N) as compared with the SARS-CoV-2 RBD, including residues that bind ACE2 (Supplementary Fig. 1b,d). To evaluate the antigenic relationships, we compared IC_50_ neutralization titres using SARS-CoV-2 and PgCoV GD nLuc live reporter viruses and a panel of commercially available and broad-spectrum SARS-CoV-2 antibodies (Fig. 5a and Supplementary Table 2). The 13 RBD-binding human Abs exhibit similar neutralization profiles against both CoVs, with less than a 10-fold difference in IC_50_ values. Under conditions where RBD class 1 and class 2 antibodies were potent neutralizers, RBD class III and S2 Ab either failed to neutralize or showed reduced potency against PgCoV GD. For example, class III antibodies, Ab COV2-2499 and COV2-2130 either exhibited a 19.4-fold reduction or failed to neutralize PgCoV, most probably associated with the R346T and Q498H mutations (Fig. 5b). Paradoxically, some RBD-specific class 1 and class 2 Abs (B38, LY-COV016, LY-COV555, ADG-2 and ADG-3) showed slightly more potent neutralization titres against PgCoV. While most commercial antibodies potently neutralized PgCoV, REGN10933 (Class 1) and 10987 (Class 3) were ~5-fold less potent. In contrast, all three NTD-binding Abs neutralized SARS-CoV-2 but not PgCoV GD, which encodes variation in the NTD Ab-binding epitopes (Fig. 5c). In addition, several COVID-19 patient convalescent sera showed similar half half-maximal inhibitory dilution (ID_50_) values (Fig. 5d,e), demonstrating that most, but not all COVID-19 patients, elicit a robust neutralizing response against PgCoV GD and SARS-CoV-2.

### PgCoV mouse models for countermeasure development

**Fig. 6:**
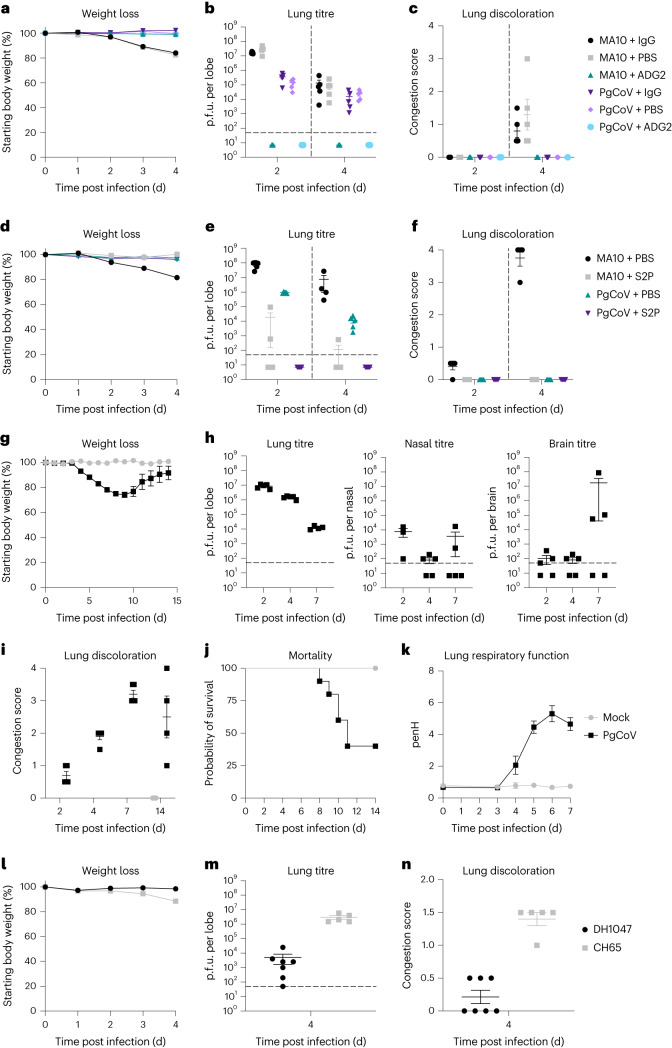
ADG-2 prophylactic and S2P vaccination studies in mice. *N* = 10 per group of BALB/c mice were treated with either ADG-2 or an isotype control antibody and then infected with PgCoV or SARS-CoV-2 MA10. PgCoV and MA10 infection (10^4^ p.f.u. per mouse) in the presence or absence of ADG-2 neutralizing antibody in BALB/c mice, *n* = 5 mice per group were collected at days 2 and 4 for titre and histological analysis. **a**, Weight loss. **b**, Lung viral titres at days 2 and 4 post infection. **c**, Lung discoloration scores. Another cohort of BALB/c mice (*n* = 10 mice per group) were vaccinated with SARS-CoV-2 S2P protein along with alum adjuvant and then challenged with PgCoV GD or mouse-adapted SARS-CoV-2 MA10 (10^4^ p.f.u. per mouse per virus), *n* = 5 mice per group were collected at days 2 and 4 for titre and histological analysis. **d**, Weight loss in PgCoV and SARS-CoV-2 MA10 challenged mice. **e**, Virus titres in the lung. **f**, Lung congestion scores of the challenged mice. We next evaluated PgCoV GD pathogenesis and DH1047 prophylaxis studies in K18-hACE2 mice, *n* = 15 mice per group. **g**, Weight loss following PgCoV GD challenge. **h**, PgCoV GD titres in the lung, nasal turbinates and brain (*n* = 5 per timepoint). **i**, Lung discoloration scores following PgCoV challenge. **j**, Percent survival following PgCoV GD challenge. **k**, Lung respiratory function (PenH) as measured by whole-body plethysmography. **l**–**n**, In another set of hACE2-K18 mice (*n* = 7 for the DH1047 group and *n* = 5 for the CH65 group), we demonstrated that the prophylactic administration of DH1047 protected against PgCoV weight loss (**l**), and reduces virus titre (**m**) and lung congestion (**n**). Data are mean ± s.d.

To evaluate PgCoV growth in standard laboratory mice, 10-month-old BALB/c mice were infected with 1 × 10^4^ p.f.u. PgCoV intranasally. In agreement with murine ACE2 receptor usage (Fig. 2), PgCoV replicated efficiently in the lungs of mice, but demonstrated little mortality, weight loss and gross lung pathology lesions as compared with SARS-CoV MA10 (Fig. 6a–d). As the pan-*Sarbecovirus* neutralizing antibody ADG-2 exhibited the highest potency against PgCoV in vitro (Fig. 5 and Supplementary Table 2), mice were treated with PBS, ADG-2 or a dengue isotype IgG control and challenged with either PgCoV or mouse-adapted SARS-CoV-2 MA10. In controls, both viruses exhibited high viral titres in the lungs on days 2 and 4 post infection, whereas ADG-2-treated mice were protected (Fig. 6a–c). Consistent with our previous finding^37^, ADG-2 significantly reduced PgCoV GD and SARS-CoV-2 MA10 weight loss, lung viral titre and gross lung discoloration (Fig. 6a–d). These data indicate that the pan-*Sarbecovirus* neutralizing Ab ADG-2 protects mice from PgCoV replication in the lung.

To evaluate whether a SARS-CoV-2 vaccine elicits cross-protection against PgCoV, we also immunized and boosted 10-week-old BALB/c mice with soluble SARS-CoV-2 Spike-2P (S2P) protein with alum adjuvant^38,39^ and demonstrated high neutralizing antibody titres in serum against both SARS-CoV-2 and PgCoV GD (Supplementary Fig. 2). After intranasal challenge with either PgCoV GD or SARS-CoV-2 MA10 at 3 weeks post boost (7 weeks post prime) (Fig. 6d–f), the S2P vaccine protected mice from SARS-CoV-2 MA10 and PgCoV GD. Little if any virus replication was noted in the lung, indicating that the SARS-CoV-2 S2P recombinant protein vaccine elicited robust cross-protection against both viruses.

Although wild-type mice provide insight into the pathogenic potential and mouse ACE2 receptor usage in vivo, the lack of clinical disease is inadequate to access human disease potential. To test a model more relevant to human populations, we evaluated PgCoV GD growth in the K18 human ACE2 mouse model, which has been repeatedly shown to progress to severe clinical disease and death following SARS-CoV-2 infection^40^. Following PgCoV GD infection, K18-hACE2-expressing mice lost weight (~25%), developed lung respiratory dysfunction, demonstrated lung discoloration and had high-titre virus replication on days 2 through 7–8 post infection (Fig. 6). Mortality rates approached 60% (Fig. 6j). To evaluate countermeasure performance, animals were then treated with hmAB DH1047, which has been shown to broadly neutralize many group 1a and 1b Sarbecoviruses and variants of concern^41^. Not only did prophylactic DH1047 administration protect from serious weight loss and clinical disease following PgCoV GD challenge, but it also significantly reduced lung discoloration scores and virus titres by about 3 logs on day 4 post infection (Fig. 6l–n). The PgCoV severe disease model in hACE2-K18 mice offers novel opportunities for countermeasure testing.

## Discussion

Coronaviruses have emerged multiple times from zoonotic reservoirs in the twenty-first century and SARS-CoV-2 mostly probably originated from strains that circulated in bats or other mammals^11,42^. Despite undersampling, SARS-CoV-2-like animal strains are indigenous to Southeast Asia and encode up to 96.8% identify with SARS-CoV-2 (ref. ^11^). One metagenomic study reported a nearly completed PgCoV genome^6^ on the basis of earlier work on smuggled pangolins confiscated in Guangdong province in March 2019^43^. Another group reanalysed the same pangolin samples and published a different full-length PgCoV genome GD^3,21^. Meanwhile, the genomes of a distant cluster of PgCoV strains GX were identified in another batch of smuggled pangolins^4,44^. The GD and GX PgCoV strains have 90.4% and 85.5% identity to the SARS-CoV-2 genome, respectively. While neither of these PgCoV strains is considered the immediate ancestor of SARS-CoV-2, PgCoV strain genetic diversity is substantially higher than previously recognized yet dramatically undersampled, increasing the probability that pangolins may have participated in the SARS-CoV-2 pandemic^45^. Unfortunately limited virus sampling, coupled with the illegal trade of these endangered species, has hampered a full characterization of the SARS-like strain diversity that circulates in pangolins^6,44,46^.

The PgCoV GD RBD preserves 13/14 hACE2 interaction sites, suggesting that recombination events between an unidentified bat *Sarbecovirus* and PgCoV GD may have contributed to SARS-CoV-2 emergence^5^. However, this hypothesis is less compelling because BANAL-52 and related group 1b strains encode near-identical RBD-ACE2 interacting residues with SARS-CoV-2 (ref. ^11^). Using pseudotypes, PgCoV spikes are reported to have a high affinity for hACE2 entry receptors^47–50^, although the in vitro and in vivo infection characteristics of the PgCoV GD strain remain unclear. In this study, we recovered a recombinant wild-type PgCoV GD strain and characterized its ACE2 receptor breadth, host range, tropism, pathogenesis in two host species, transmissibility and antigenicity. Supporting structural studies^47^, the PgCoV GD strain and other closely related clade 1b SARS-CoV-2 zoonotic strains^11^ may efficiently engage a large number of mammalian ACE2 receptors for docking and entry, replicate efficiently in multiple mammalian host species, and cause clinical disease and mortality in k18-hACE2-expressing mice, but not in standard laboratory mice. Importantly, PgCoV GD replicates efficiently in primary human epithelial cells from the proximal to distal human lung and undergoes airborne transmission between hamster pairs, albeit inefficiently as compared with SARS-CoV-2 Wuhan.

Current models of coronavirus emergence acknowledge the existence of zoonotic strains that encode spike glycoproteins with broad orthologue receptor specificities (Supplementary Fig. 3)^19,20,51^. For SARS-CoV and MERS-CoV, intensive farming practices involving civets and camels were critical for virus amplification and evolution, leading to repeat introductions into human populations^52^. As with many zoonosis, the reservoir species and index case for the SARS-CoV-2 pandemic remains elusive, although pangolins and racoon dogs remain suspects. Pangolins, which harbour distinct SARS-CoV-2-related strains^2–6^, are an endangered solitary species but are valuable as illegal trade commodities. On the basis of the broad ACE2 receptor usage of PgCoV GD, documented airborne transmission potential and efficient growth in primary nasal airway epithelial cells, we suggest that individual pangolins, or perhaps some other rare wildlife species, was productively infected and served as a nearly untraceable pass-through species that transmitted virus to humans. Such rare events might be enhanced if early human cases were immunosuppressed, potentially generating complex mutational variants during persistent infections^53–55^ (Supplementary Fig. 3).

Although speculative, a variety of data support this hypothesis. Recombinant PgCoV GD and SARS-CoV-2 share a similar host range^44,56^ and use pangolin, human and many other mammalian ACE2 receptors for docking and entry^47,50^. PgCoV, but not SARS-CoV WA1, can efficiently utilize the murine ACE2 receptor for entry and can infect mice^18^. However, several SARS-CoV-2 variants of concern (VOC) quickly evolved the N501Y mutation that enhanced growth in mice^17,57^. In contrast, the PgCoV Q498H RBD mutation probably influenced rodent susceptibility, as a similar Q498Y mutation in the SARS-CoV-2 WA1 strain conferred mACE2 receptor usage and growth in the laboratory mouse^18,44^. Humans have transmitted SARS-CoV-2 to other mammals, such as minks, tigers and American white-tailed deer^58–60^. As mink to human and deer to human transmission is documented, new animal reservoirs are emerging that support the evolution and transmission of new SARS-CoV-2 variants^61–64^. *Sarbecovirus* infections in pangolins and other susceptible mammals (for example, racoon dogs, civets) are understudied. One recent report noted that among the 334 pangolins confiscated in Malaysia from 2009 to 2019, none contained CoV RNA, while another study in confiscated pangolins showed 4/163 infected with virus^45^. Clearly, pangolins are not a heavily colonized reservoir species but rather, could serve as rare solitary hosts for zoonotic SARS-CoV-2-like viruses. The extensive overlap in potential host range suggests that the SARS-CoV-2 and PgCoV GD strains probably emerged from a common source, most probably generalists in bats. Given the close homology between PgCoV GD RBD and the different Banal strains (Supplementary Fig. 1d), our data support earlier hypotheses that Sarbecoviruses in pangolins present an emerging threat to human health (Supplementary Fig. 3).

Protease cleavage sites in the S glycoprotein play critical roles in coronavirus entry, pathogenesis and transmission. In contrast to SARS-CoV-2, PgCoV GD and related strains lack the S1/S2 RRAR polybasic furin cleavage site (682–686). Despite this deficiency, PgCoV GD infectivity and early growth phenotypes were more efficient that SARS-CoV-2 in cell lines overexpressing furin. Of interest, another furin cleavage site (K814) in SARS-CoV-2, RaTG13 and PgCoV GD regulated S2 into S2’ cleavage and infectivity^27,28^. Removal of the SARS-CoV-2 S1/S2 polybasic cleavage site (682–686) also enhanced virus titre stability and growth in Vero cells in vitro. Interpretation of loss-of-function studies is complicated by off-target effects on spike protein/structure–function and unknown epistatic interactions^65^. For example, the SARS-CoV-2 S1/S2 furin cleavage site resides within a disordered region flanked by serine residues that can be phosphorylated by proline-directed and basophilic kinases to inhibit proteolytic cleavage^66^. In addition, an upstream O-linked glycosylation site also regulates cleavage efficacy^67,68^. Loss-of-function studies deleting the RRAR furin cleavage site or upstream QTQTN residues that contain the O-linked glycosylation site resulted in virus mutants with reduced in vitro replication in airway epithelial cells and in vivo pathogenicity and/or transmission^14,69^. However, these loss-of-function mutations also attenuated cleavage at overlapping S1/S2 protease sites such as TMPRSS2, complicating data interpretation^13^. In the absence of the S1/S2 polybasic furin cleavage site, PgCoV GD replicated as efficiently as SARS-CoV-2 in primary human nasal airway epithelial and large bronchial airway epithelial cells. In contrast, PgCoV GD replication efficiency in AT2 organoids ranged from equally efficient to less efficient than SARS-CoV-2 in a donor-dependent manner. The 2003 SARS-CoV strain, which lacks an S1/S2 furin cleavage site, replicated efficiently in primary human epithelial cells, was efficiently transmitted between civets, raccoon dogs and humans, and caused ~10% mortality rates in humans^70^. As humans living and working near bat hibernacula experience occasional bat SARS-like CoV infections^71^, it appears that Sarbecoviruses use multiple protease-dependent pathways to ensure efficient transmission.

Antigenic variation in spike (~3%) has reduced vaccine and therapeutic antibody performance against SARS-CoV-2^72,73^. Although the PgCoV GD spike is ~10% different from SARS-CoV-2 spike, the virus encode many conserved neutralizing epitopes in the RBD but not in the NTD domain. Importantly, COVID-19 convalescent patient sera and many commercial therapeutic antibodies effectively neutralized PgCoV in vitro. Unlike another PgCoV strains^44^, PgCoV GD caused lethal outcomes in K18-hACE2 mice, but not in wild-type mice. Using these models, the pan-*Sarbecoviru*s mAB ADG-2 (ref. ^37^), hmAB DH1047 and SARS-CoV-2 S2P recombinant protein vaccines protected animals from PgCoV GD replication and disease. Thus, the current COVID-19 vaccines and immunotherapeutics should protect humans from PgCoV GD infection. Although the mechanism is probably complex, competition studies in primary human cells demonstrated reduced PgCoV GD fitness as compared with SARS-CoV-2.

Virus discovery is critical for One Health, policy, global health preparedness and the development of broadly effective countermeasures. Field collections, coupled with virus recovery in cultured cells and animals oftentimes under Biosafety Level 2 (BSL2) conditions, are historically used to identify viruses in the natural world^74,75^. Today, high-throughput sequencing technologies and the use of molecular approaches and synthetic genomics to recover recombinant viruses from wild-type genome length sequences have altered this historic paradigm^76^. The PgCoV GD strain represents a replica of a wild-type PgCoV strain circulating in nature and was isolated by researchers wearing protective clothing in a highly controlled BSL3 environment. No genetic approaches were implemented to alter the intrinsic biology and pathogenesis of the virus. Lacking ORF7, PgCoV GD derivatives encoding reporter genes such as GFP and nLUC were generated to provide high-throughput diagnostic reagents to understand virus tropism, neutralization and countermeasure performance. The paradigm allows for the recovery of a single, targeted virus from a virologically complex field sample in a safe, controlled BSL3 facility, thereby reducing the risk of the inadvertent recovery of some unidentified contaminant^41,57,77^. Although the PgCoV GD strain provides an optimal model to evaluate the impact of a furin cleavage site at the S1/S2 border on replication, pathogenesis and transmission, we urge constraint as such studies should require transparent, independent and rigorous review^57^.

## Methods

### Biosafety

This research complies with all relevant ethical regulations, and study protocols were approved by institutional boards and committees. Synthetic reconstruction of the authentic, wild-type Pangolin Coronavirus GD virus strain was based on its published sequence and undertaken with the approval of the Institutional Biosafety Committee of the University of North Carolina at Chapel Hill (Application no. 80022). All constructs were handled under proper biosafety level conditions as specified by the institutional approval, with reconstructed virus handled exclusively at biosafety level 3, with personnel wearing full-body personal protective equipment and HEPA-filtered respiratory protection. Current US Gain-of-Function (GOF) regulations note that wild-type pathogens that are circulating in or have been recovered from nature are not enhanced potential pandemic pathogens (PPPs), regardless of their pandemic potential.

### Cells and viruses

Simian kidney cell line Vero-E6 (ATCC, CRL1586) was maintained in Eagle’s minimum essential medium (Gibco) supplemented with 10% fetal calf serum (FBS, Hyclone). A clonal furin-overexpressing Vero cell line was generated by using the Sleeping Beauty Transposon System as previously described by our group^29^. Briefly, the plasmid encoding the furin gene was transfected into the Vero cells, and the cells were selected by appropriate antibiotics. A549-ACE2 cells were maintained in Dulbecco’s modified Eagle medium (DMEM, Gibco) with 10% FBS. Using the Sleeping Beauty Transposon System^34^, we expressed a panel of ACE2 orthologues from the bat *Rhinolophus affinis* (GenBank, MT394215.1), the bat *R. alcyone* (GenBank, ALJ94035), camel (GenBank, XM_010993415.2), cat (GenBank, AB211997), cow (GenBank, XM_024987850.1), ferret (GenBank, NM_001310190), horse (GenBank, XM_001490191.5), rabbit (XM_002719845.3), hamster (GenBank, XM_005074209.3), dog (GenBank, NM_001165260.1), pangolin (GenBank, XM_017650263.2), mouse (GenBank, NP_001123985.1), mink (GenBank, XP_044091952) and civet (GenBank, AY881174) in non-permissive DBT cells. All ACE2 constructs included a FLAG tag at the C terminus, except the mouse and *R. alcyone* receptors. After selection with puromycin or gentamycin (mink, *R. alcyone*, mouse), ACE2 expression was confirmed by western blot analysis using a polyclonal ACE2 antiserum.

The primary human pulmonary cell cultures were purchased from the UNC Marsico Lung Institute cell bank. The generation of primary human pulmonary cell cultures was described previously^22^. Primary HNE cells, human bronchial epithelial LAE cells and bronchiolar (small airway epithelial (SAE)) cells were isolated from freshly excised normal human tissues obtained from transplant donors under UNC Institutional Review Board (IRB)-approved protocol (no. 03-1396) and cultured in air–liquid interface (ALI) media, as previously described^22,78^. The age and gender of the donors included males and females 21–70 years of age.

The molecular clone-derived SARS-CoV-2 WT and nLuc viruses were generated as previously described^18,22^. Briefly, genomic cDNA sequences of these viruses were ligated in vitro and subjected to T7 transcription. The transcribed RNA was electroporated into Vero cells. SARS-CoV-2-∆PRRA was generated by deleting the four residues ‘PRAA’ coding sequence from the SARS-CoV-2 WT infectious cDNA clone. All work was conducted in a high-containment BSL3 laboratory and personnel wore powered air purifying respirator (PAPR), Tyvek suits, aprons and booties, and were double gloved.

#### SARS-CoV-2-related pangolin-CoV antiviral assay

The antiviral activity of RDV, EIDD and nirmatrelvir against SARS-CoV-2-related pangolin-CoV GD (MP789) was measured in a A549-hACE2 cell line-based assay. The human cell line, A549-hACE2, was maintained in DMEM (Gibco), 20% fetal bovine serum (Hyclone) and 1X antibiotic-antimycotic (A.A., Gibco). At 24 h after plating 2 × 10^4^ cells per well, fresh medium was added. In triplicate, cells were exposed to serial dilutions of compound and control compounds (RDV, EIDD, nirmatrelvir) in ‘infection medium’ (that is, modified growth medium similar to that above but with 5% FBS) and immediately infected for 1 h with SARS-CoV-2-related pangolin-CoV nLUC added at 800 p.f.u. per well. Virus was then removed, and cells were rinsed once and infection medium containing dilutions of drug or vehicle was added. At 24 h post infection, virus replication was measured by nLUC assay (Promega) and then read on a Promega Glomax plate reader (Promega). The IC_50_ value was defined in Graphpad Prism 9.0 (Graphpad) as the concentration at which there was a 50% decrease in viral replication, using non-infected wells (100% inhibition) and infected and vehicle-treated wells (0% inhibition) as controls. The experiment was repeated once.

### Generation of infectious cDNA clones for PgCoV GD1

Seven cDNA fragments covering the entire genome of the PgCoV GD strain (GISAID accession no. EPI_ISL_410721) were chemically synthesized by Bio Basic. Fragments were cloned into vector plasmid pUC57, and junctions were divided by non-palindromic sites BsaI (GGTCTCN^NNNN), BsmBI (CGTCTCN^NNNN) or SapI (GCTCTTCN^NNN) with unique 3- or 4-nucleotide cohesive ends. The cohesive ends in each fragment are indicated in Fig. 1a. To assist the replication of the full-length viral genome, we introduced a T7-promoter sequence into the 5’-end of fragment A and a 25 nt poly-A tail into the 3’-end of the fragment G. Each fragment was verified by Sanger sequencing. To enhance the efficiency of recovering the PgCoV in the cell culture, an sgRNA-N construct encoding a 5’ leader sequence, an N gene, a 3’ UTR and a 25 nt poly-A tail was assembled downstream of a T7 promoter. Two reporter viruses containing a GFP or an nLuc gene were generated by replacing part of the ORF7a gene with the reporter genes.

### Recovery of recombinant viruses

Seven genomic cDNA fragments were digested with appropriate restriction enzymes, resolved in 1% agarose gels, excised and purified using a QIAquick Gel Extraction kit (Qiagen). A full-length genomic cDNA was obtained by ligating seven fragments in an equal molar ratio using T4 DNA ligase (NEB). The full-length PgCoV genomic RNA or sgRNA-N was synthesized using the T7 mMESSAGE T7 transcription kit (Thermo Fisher) at 32 °C for 5 h. The RNA transcripts were then mixed and electroporated into Vero-E6 cells at 450 V and 50 µF using a Gene Pulser II electroporator (Bio-Rad). The cells were cultured as usual in the medium for 3–4 d.

### nLuc virus entry assay

Monolayers of Vero-E6 and Vero-furin cells were cultured in black-walled 96-well plates (Corning 3904) overnight. The cells were infected with SARS-CoV-2-nLuc or PgCoV-nLuc viruses at an MOI of 1. After incubation for 1 h, the inoculum was removed, the cells were washed two times with PBS and maintained in DMEM containing 5% FBS and the mixture of SARS-CoV-2 nAbs REGN10933 and REGN10987 at a concentration of 1,000 times the IC_50_ against SARS-CoV-2. After incubation at 37 °C for 8 h, viral entry was quantified by measuring nLuc activity using a Nano-Glo luciferase assay system (Promega) according to manufacturer specifications.

### Virus entry assay in ACE2 orthologue-expressing cells

Monolayers of ACE2 orthologue-expressing A549 cell lines were cultured in black-walled 96-well plates (Corning 3904) overnight. The cells were infected with a PgCoV-GFP virus at an MOI of 0.5. For fluorescence imaging, the cells were seeded in clear-walled 96-well plates (Corning, 3598) and images captured in a Nikon fluorescence scope. Experiments were repeated twice.

### Western blot analysis of spike protein cleavage

Extracellular PgCoV and SARS-CoV-2 virions were collected from Vero or Vero-furin cells. Samples were lysed with modified RIPA buffer and inactivated at 98 °C. Protein samples were electrophoresed in 4–20% continuous SDS–PAGE gel (Bio-Rad) and transferred onto a PVDF membrane (Bio-Rad). Spike proteins of both viruses were probed using a mAb targeting a conserved region in S2 (Abcam, ab272504), and the N protein was probed using a mouse antiserum produced in our laboratory. The western blot images were captured and quantified using the Thermo Fisher iBright imaging system and software.

### Northern blot analysis

Vero-E6 cells were infected with SARS-CoV-2 isolate, icPgCoV-WT, icPgCoV-GFP, icPgCoV-nLuc or mock at an MOI of 1. At 24 h post infection, we extracted the total cellular RNA using TRIzol reagent (Thermo Fisher). Poly-A-containing messenger RNA was isolated from the total RNA using an Oligotex mRNA mini kit (Qiagen). Messenger RNA (0.6–0.7 μg) was separated on an agarose gel and transferred to a BrightStar-Plus membrane using a NorthernMax-Gly kit (Invitrogen). Blots were hybridized with a biotin-labelled oligomer (5’-BiodT/GGCTCTGTTGGGAATGTTTTGTATGCG/BiodT-3’), then detected with a chemiluminescent nucleic acid detection module (Thermo Fisher) using the iBright western blot imaging system (Thermo Fisher).

### PgCoV and SARS-CoV-2 neutralization assay

Vero-E6 cells were plated at 20,000 cells per well in black-walled 96-well plates (Corning 3904). Human serum samples were tested at a starting dilution of 1:40 and mAb samples were tested at a starting concentration of 30 to 0.1 μg ml^−1^ and were serially diluted 3-fold for up to eight dilution spots. Diluted antibodies and sera were then mixed with 200 p.f.u. per well of PgCoV-nLuc or SARS-nLuc virus and the mixtures were incubated at 37 °C with 5% CO_2_ for 1 h. Following incubation, growth media were removed and virus–antibody mixtures were added to the cells in duplicate. Virus-only controls were included in each plate and all samples were run in duplicate. Following infection, plates were incubated at 37 °C with 5% CO_2_ for 48 h. After the 48 h incubation, cells were lysed and luciferase activity was measured via a Nano-Glo luciferase assay system (Promega) according to manufacturer specifications. Neutralization titres were defined as the sample dilution at which a 50% reduction in the relative light units (RLU) was observed relative to the average of the virus control wells.

### Hamster infection, tissue collection and transmission studies

Hamster studies were performed in accordance with University of Wisconsin-Madison Institutional Animal Care and Use Committee (IACUC) protocol no. V006426. Syrian hamsters (females, 4–6 weeks old) were purchased from Envigo and allowed to acclimate for a minimum of 3 d at BSL3 agriculture containment at the Influenza Research Institute (University of Wisconsin). Hamsters were infected with 10^3^ p.f.u. of PgCoV GD or a D-form SARS-CoV-2 strain NCGM02 intranasally under isoflurane anaesthesia. At the indicated timepoints, a subset of hamsters was euthanized by deep anaesthesia using isoflurane inhalation and cervical dislocation, and tissue samples were collected for virus titre. No animals were excluded from the study and animals were randomly assigned to groups. Sections were stained and scored by pathologists blinded to the experimental groups.

To evaluate indirect virus transmission between hamsters, groups of hamsters (*n* = 6 per group) were infected with 10^3^ p.f.u. of PgCoV or SARS-CoV-2 viruses intranasally under isoflurane anaesthesia. Infected animals were placed in specially designed cages inside an isolator unit^34,35^. After 24 h, naïve hamsters were placed on the other side of the cage, with 5-cm separation by a double-layered divider to allow free airflow. The isolator unit provided one-directional airflow; therefore, the infected hamsters were placed in the front of the isolator unit. Metal shrouds were placed over the cages so that only the front and back of the cage was open. Nasal washes were collected at 3-d intervals for the infected hamsters and 2-d intervals for the exposed animals starting on day 2 after infection or exposure.

### Mouse studies

Mouse studies complied with all relevant ethical regulations and were performed after UNC IACUC protocol no. 20-074 review and approval. For the mAB ADG-2 prophylactic study, 10-week-old female BALB/c mice (Envigo, 047) were inoculated with 200 µg of indicated antibodies intraperitoneally 12 h before infection. For the SARS-2 S2P vaccine study, 10-week-old female BALB/c mice (Envigo, 047) were primed with 500 μg of S2P protein in 10 µg aluminum hydroxide suspension intramuscularly and boosted 4 weeks later. ADG-2-injected or S2P-vaccinated mice were anaesthetized with a mixture of ketamine/xylazine and infected with 10^4^ p.f.u. of either PgCoV or mouse-adapted SARS-CoV-2 (MA10). Alternatively, hACE2-K18 mice were prophylactically treated with mAB DH1047 at 200 μg intraperitoneally or with control isotype and infected with PgCoV GD. Mice were monitored daily for clinical signs of disease (weight loss) and mortality. At indicated timepoints, mice were euthanized via isoflurane overdose and samples for titre and histopathological analyses were collected. Titre samples were collected in PBS with glass beads and stored at −80 °C until further processing; viral lung, nasal or brain titres were determined via plaque assay as described above. Histological samples were stored in 10% formalin at 4 °C for 7 d and formalin was replaced with fresh 10% formalin before removal out of the BSL3. No animals were excluded from the study and animals were randomly assigned to groups.

### Whole-mount immunostaining and imaging

Primary airway cell cultures and AT2 organoids were fixed twice for 30 min in 4% formaldehyde in PBS, and washed and stored in PBS. The viral signals were stained by a mouse antiserum against SARS-CoV-2 N protein (1:500 dilution) using species-specific secondary antibodies as previously described^22^. The HNE and LAE cultures were also imaged for α-tubulin (Millipore MAB1864; 3 μg ml^−1^), MUC5AC (Thermo Scientific 45M1; 4 μg ml^−1^), MUC5B (polyclonal rabbit against a MUC5B peptide (MAN5BII), 1:1,000) and CCSP (Sigma 07-623; 1:2,000) as indicated. Filamentous actin was localized with phalloidin (Invitrogen, A22287) and DNA with Hoechst 33342 (Invitrogen). An Olympus FV3000RS confocal microscope in Galvo scan mode was used to acquire 5-channel *Z* stacks by the 2-phase sequential scan. Representative stacks were acquired with a ×60 oil objective (*XYZ* = 212 μm × 212 μm × ~25 μm) and shown as *Z*-projections or single-slice, *XZ* cross-sections to distinguish individual cell features to characterize the infected cell types. A ×20 objective was used to acquire 2D, single-channel, apical snapshots of nine fields (636 μm × 636 μm; combined area of 3.64 mm^2^), selected in evenly spaced grids across each sham-infected donor culture, and ImageJ was used to measure the relative apical culture surface covered by multiciliated cells.

### Statistical analysis

No statistical methods were used to pre-determine sample sizes, but our sample sizes are similar to those reported in previous publications from our group (18, 19, 20, 22). Technical and biological replicates are described in the figure legends. Data distribution was assumed to be normal, but this was not formally tested. For analysis of PgCoV and SARS-CoV-2 nLuc virus entry (Fig. 1e), we applied an unpaired *t*-test. For COVID-19 patient sera cross-neutralization against PgCoV and SARS-CoV-2 (Fig. 5e), we applied paired *t*-test. For analysis of mouse studies, the comparison of weight-change curves was performed using a repeated measurements two-way ANOVA with Tukey’s post hoc test using Prism v.9.0 (GraphPad). Results of transmission studies were compared using Fischer exact test, while paired *t*-test and unpaired *t*-test were used in Figs. 5e and 1e, respectively.

### Reporting summary

Further information on research design is available in the Nature Portfolio Reporting Summary linked to this article.

### Supplementary information

Supplementary Information Supplementary Figs. 1–3, and Tables 1 and 2.

Reporting Summary

### Source data

Source Data Fig. 1 Unprocessed western blot images for Fig. 1d.

Source Data Fig. 1 Unprocessed northern blot images for Fig. 1c.

Source Data Fig. 2 Unprocessed western blot images for Fig. 2b.

## Data Availability

The experiment data that support the findings of this study are available from the corresponding author upon request. In addition, we will provide Biodefense and Emerging Infections Research Resources Repository (BEI) with the recombinant wild-type PgCoV virus, including indicator viruses encoding nLUC and GFP. Users would be requested to demonstrate approval for receiving the virus from their institutional biosafety group and work with the virus under Biosafety Level 3 (BSL3) conditions. Requests for the PgCoV molecular clone should be directed to the corresponding author and will require a description of the planned experimental studies listed under a Material Transfer Agreement. As experiments of concern could be performed with this construct, we will review the request for the molecular clone with NIH for feedback and approval before providing the reagent. The materials transfer timeline shall be determined by the appropriate time for reviewing the documentations by the University of North Carolina and NIH. Source data are provided with this paper.
