# Supplementary material for: Host range, transmissibility and antigenicity of a pangolin coronavirus
Source: Nat Microbiol. 2023 Sep 25;8(10):1820–33. doi: 10.1038/s41564-023-01476-x (PMC10522490; doi:10.1038/s41564-023-01476-x)
Supplement: Supplementary file 1 — Supplementary Figs. 1–3, and Tables 1 and 2. [file 41564_2023_1476_MOESM1_ESM.pdf]

nature microbiology

Article

https://doi.org/10.1038/s41564-023-01476-x

Host range, transmissibility and antigenicity of a pangolin coronavirus

In the format provided by the authors and unedited

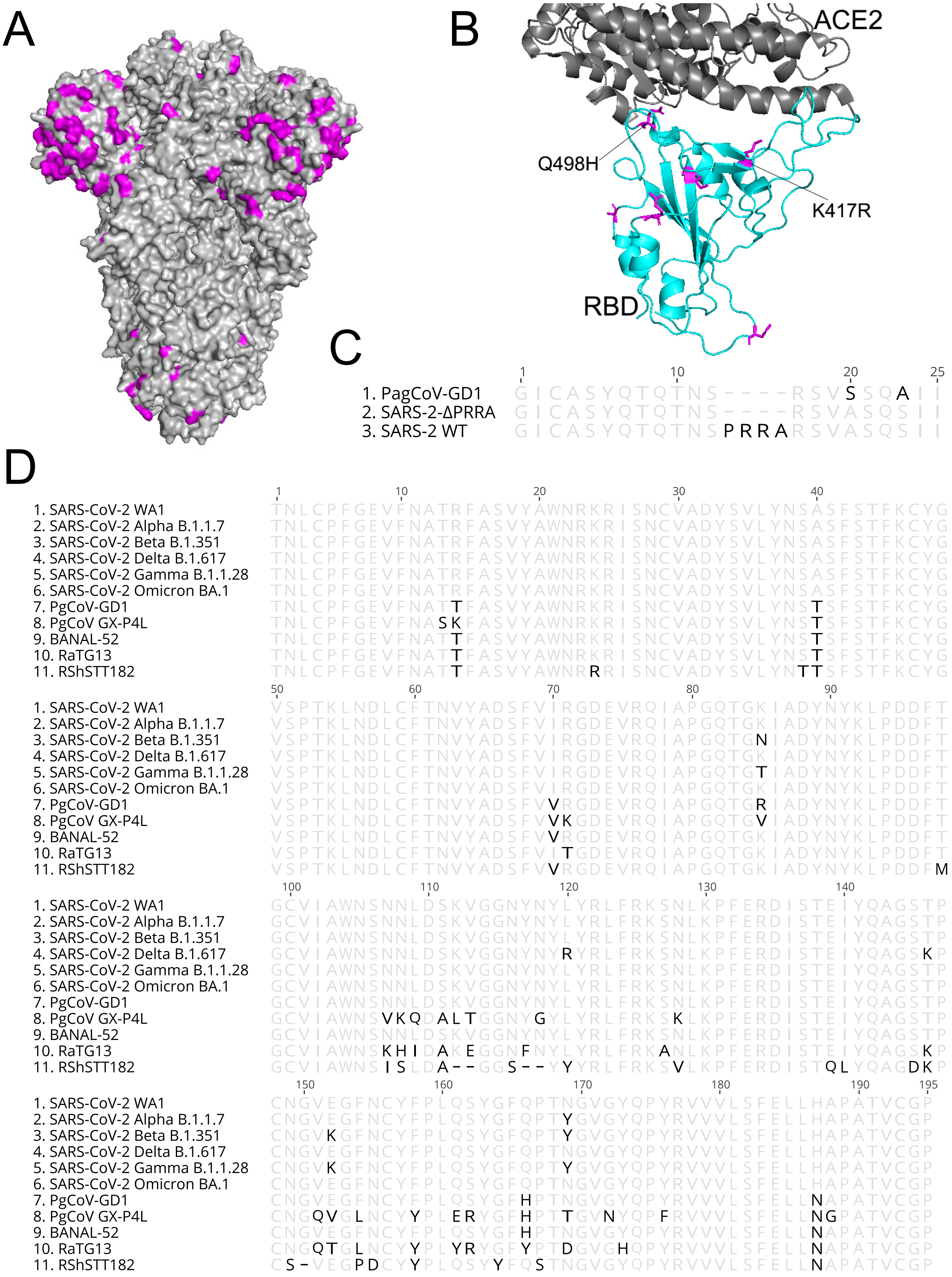

Supplementary Figure 1. PgCoV Spike Variation. Panel A. Annotation of amino acid variations (magenta) between PgCoV and SARS-CoV-2 in the spike trimer (PDB ID: 6vsb); Panel B. six amino acid variations (magenta) within the RBD (cyan), comparing to SARS-CoV-2. The two substitutions located in the ACE2 (grey) binding interfaces are Q498H and K417R. (PBD ID: 6vw1) Panel C. Alignments of amino acids around S1/S2 junctions between PgCoV, SARS-2 WT, and SARS-2ΔPRRA. Panel D. Alignment of RBD between SARS-CoV-2 WA1 strain, variants of concerns, PgCoV strains, and several closely related bat Sarbecoviruses. The PgCoV RBD spans residues 327-520 while the S1/S2 furin cleavage site (ΔPRRA) would have resided between residues S681 and R682, when comparing full length PgCoV and SARS-CoV-2 spike sequences.

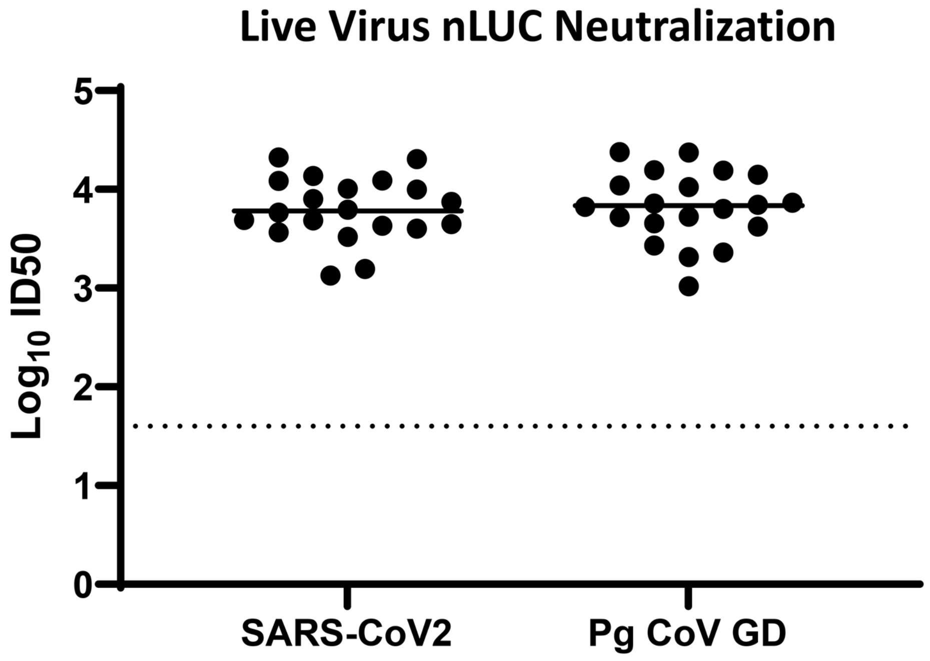

Supplementary Figure 2. Recombinant S2P + Alum Neutralization Antibody Titers. Mice (n=20) were primed and boosted with SARS-CoV-2 S2P + alum. Prior to challenge, serum was collected and evaluated for neutralization using SARS-CoV-2 D614G and PgCoV GD nLUC viruses and reported as Log10 LD50. Data are presented as mean values ± SD.

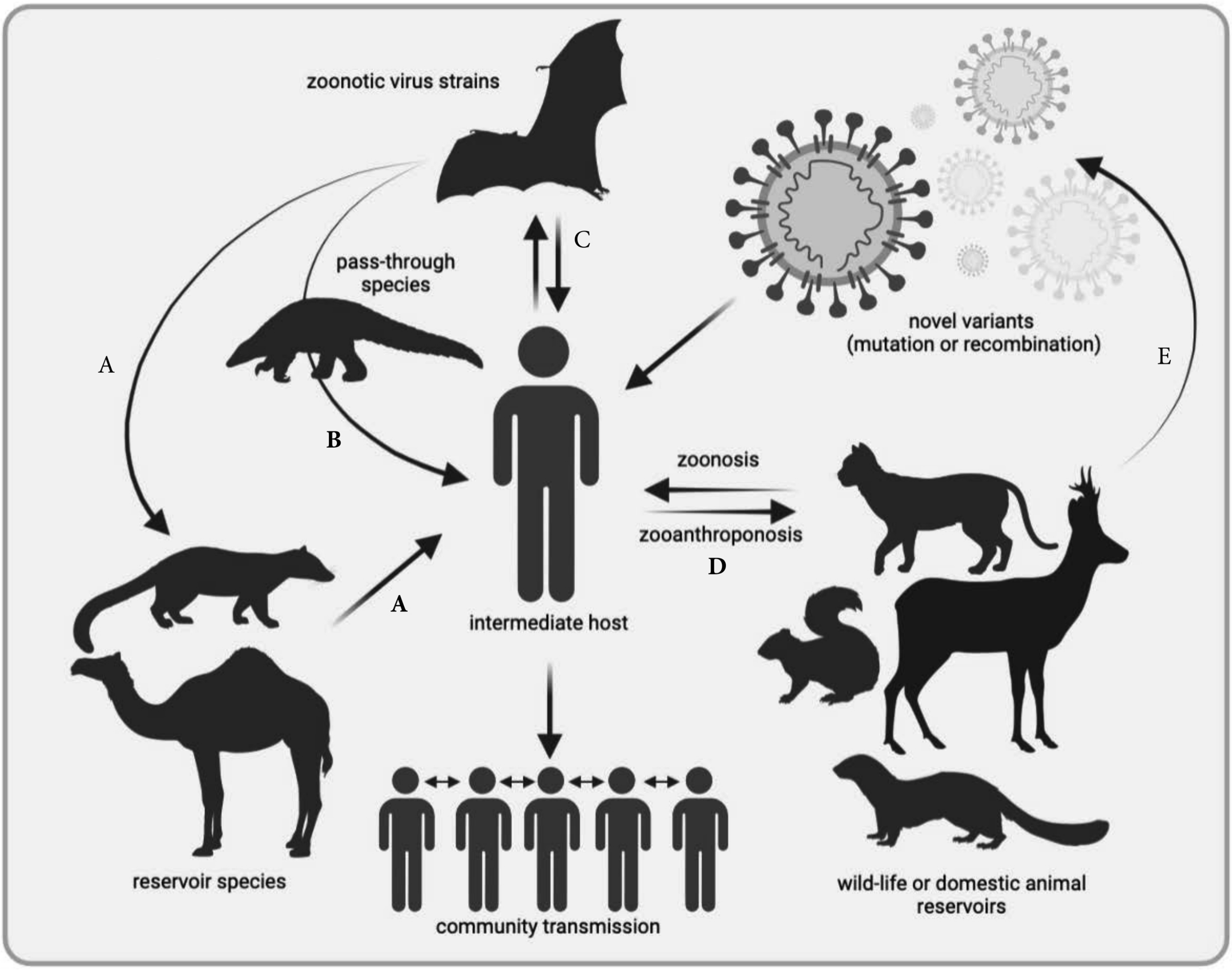

Supplementary Figure 3. Models for Emerging Coronavirus Cross Species Transmission. The classic model for coronavirus emergence involves transmission of zoonotic virus strains that circulate broadly and evolve in an intermediate reservoir species before spreading to human hosts (A). However, alternative pathways for zoonotic virus introductions include rare pass through species that are rare but are in close proximity and can directly transmit to human hosts (B) or via direct introduction into humans from bats (C). By zooanthroponosis (D), human hosts also function as intermediate hosts for the transmission and maintenance of virus in other species, which can drive novel evolutionary trajectories and potentially reintroduce virus variants into human hosts (E).

Supplementary Table 1. Lung Pathology* and IHC Score# of hamsters infected by PgCoV.

Sample ID	Perivascular inflammation	Bronchial/ bronchiolar epithelial degeneration /necrosis	Bronchial/bronchiolar inflammation	Alveolar inflammation	Total Pathology Score (H&E)	IHC score
PgCoV Lung 1	1	1	2	2	6	2
PgCoV Lung 2	1	1	1	1	4	1
PgCoV Lung 3	0	1	1	3	5	2
PgCoV Lung 4	1	0	1	1	3	0
PgCoV Lung 5	1	1	1	1	4	1
PgCoV Lung 6	2	1	1	2	6	2

- *A 5-point scoring system was utilized (0-within normal limits, 1-mild, 2-moderate, 3-marked, 4-severe) in H&E sections.
- #A 4-point scoring system was utilized (0- none, 1-minimal, 2-moderate, 3-abundant signals) in IHC sections.
- A total pathology score was calculated for each hamster by adding the individual histopathological feature scores.
- A maximum total pathology score of 16 is possible for an individual hamster. At 4DPI, all hamsters exhibited typical microscopic lesions of broncho-interstitial pneumonia.
- Individual pathology scores varied from 3/16 to 6/16 and were determined in collaboration with a certified pathologist at Histowiz (www.histowiz.com).

Supplementary table 2. IC_{50} values of A panel of SARS-CoV-2 antibodies cross neutralization against PgCoV and SARS-CoV-2

		IC_{50} (µg/mL)	
**SARS-2 Abs**	**Binding domain**	**Anti-PgCoV**	**Anti-SARS-2**
4A8	NTD	>30	0.027
COV2-2489	NTD	>10	0.5469
COV2-2676	NTD	>10	0.4678
COV2-2164	S2	>10	>10
B38	RBD (Class I)	0.94	7.15
S309	RBD (Class III)	0.138	0.143
LY-COV016	RBD (Class I)	0.086	0.126
REGN10933	RBD (Class I)	0.108	0.023
REGN10987	RBD (Class III)	0.062	0.0097
LY-COV555	RBD (ClassI/III)	0.0091	0.042
ADG-2	RBD (Class I)	0.00335	0.04489
ADG-1	RBD	0.0285	0.045
ADG-3	RBD	0.0136	0.1053
COV2-2196	RBD (Class I/II)	0.1444	0.0558
COV2-2130	RBD (Class III)	>10	0.1905
COV2-2479	RBD (Class II)	0.06513	0.06313
COV2-2499	RBD (Class III)	1.429	0.07356
COV2-2832	RBD (Class II)	0.0383	0.02279
COV2-2082	RBD (Class I)	0.041	0.05002
