# Supplementary material for: Host range, transmissibility and antigenicity of a pangolin coronavirus
Source: Nat Microbiol. 2023 Sep 25;8(10):1820–33. doi: 10.1038/s41564-023-01476-x (PMC10522490; doi:10.1038/s41564-023-01476-x)
Supplement: Supplementary file 2 — Reporting Summary [file 41564_2023_1476_MOESM2_ESM.pdf]

nature portfolio

Corresponding author(s): Ralph S Baric

Last updated by author(s): May 24, 2023

Reporting Summary

Nature Portfolio wishes to improve the reproducibility of the work that we publish. This form provides structure for consistency and transparency in reporting. For further information on Nature Portfolio policies, see our Editorial Policies and the Editorial Policy Checklist.

Statistics

For all statistical analyses, confirm that the following items are present in the figure legend, table legend, main text, or Methods section.

n/a	Confirmed	
☐	☒	The exact sample size (*n*) for each experimental group/condition, given as a discrete number and unit of measurement
☐	☒	A statement on whether measurements were taken from distinct samples or whether the same sample was measured repeatedly
☐	☒	The statistical test(s) used AND whether they are one- or two-sided<br>*Only common tests should be described solely by name; describe more complex techniques in the Methods section.*
☒	☐	A description of all covariates tested
☐	☒	A description of any assumptions or corrections, such as tests of normality and adjustment for multiple comparisons
☐	☒	A full description of the statistical parameters including central tendency (e.g. means) or other basic estimates (e.g. regression coefficient) AND variation (e.g. standard deviation) or associated estimates of uncertainty (e.g. confidence intervals)
☒	☐	For null hypothesis testing, the test statistic (e.g. *F*, *t*, *r*) with confidence intervals, effect sizes, degrees of freedom and *P* value noted<br>*Give P values as exact values whenever suitable.*
☒	☐	For Bayesian analysis, information on the choice of priors and Markov chain Monte Carlo settings
☒	☐	For hierarchical and complex designs, identification of the appropriate level for tests and full reporting of outcomes
☒	☐	Estimates of effect sizes (e.g. Cohen's *d*, Pearson's *r*), indicating how they were calculated

Our web collection on statistics for biologists contains articles on many of the points above.

Software and code

Policy information about availability of computer code

Data collection	N//A
Data analysis	N/A

For manuscripts utilizing custom algorithms or software that are central to the research but not yet described in published literature, software must be made available to editors and reviewers. We strongly encourage code deposition in a community repository (e.g. GitHub). See the Nature Portfolio guidelines for submitting code & software for further information.

Data

Policy information about availability of data

All manuscripts must include a data availability statement. This statement should provide the following information, where applicable:

- Accession codes, unique identifiers, or web links for publicly available datasets
- A description of any restrictions on data availability
- For clinical datasets or third party data, please ensure that the statement adheres to our policy

All relevant data are included in this article.Reagents and resources are available upon request from the corresponding author and with a material transfer agreement. Source data are provided with this paper.

Research involving human participants, their data, or biological material

Policy information about studies with human participants or human data. See also policy information about sex, gender (identity/presentation), and sexual orientation and race, ethnicity and racism.

Reporting on sex and gender	N/A, the primary human airway cells were purchased form UNC cell bank and we were blinded about the demographic information about the tissue donors.
Reporting on race, ethnicity, or other socially relevant groupings	N/A
Population characteristics	N/A
Recruitment	N/A
Ethics oversight	N/A

Note that full information on the approval of the study protocol must also be provided in the manuscript.

Field-specific reporting

Please select the one below that is the best fit for your research. If you are not sure, read the appropriate sections before making your selection.

☒ Life sciences ☐ Behavioural & social sciences ☐ Ecological, evolutionary & environmental sciences

For a reference copy of the document with all sections, see nature.com/documents/nr-reporting-summary-flat.pdf

Life sciences study design

All studies must disclose on these points even when the disclosure is negative.

Sample size	The sample size info are indicated in different figure legends and method section. The sample size were determined to ensure enough replicates for statistical analysis.
Data exclusions	No data were excluded
Replication	The replications are indicated in different figure legends and method section.
Randomization	The randomization info are indicated in different figure legends and method section. For animal studies, individual animal was randomly grouped. For in vitro study, cell cultures were mixed and randomly allocated into different culture containers for experiments.
Blinding	Investigators were blinded when collecting and analyzing the data.

Reporting for specific materials, systems and methods

We require information from authors about some types of materials, experimental systems and methods used in many studies. Here, indicate whether each material, system or method listed is relevant to your study. If you are not sure if a list item applies to your research, read the appropriate section before selecting a response.

Materials & experimental systems

n/a	Involved in the study	
☐	☒	Antibodies
☐	☒	Eukaryotic cell lines
☒	☐	Palaeontology and archaeology
☐	☒	Animals and other organisms
☒	☐	Clinical data
☒	☐	Dual use research of concern
☒	☐	Plants

Methods

n/a	Involved in the study	
☒	☐	ChIP-seq
☒	☐	Flow cytometry
☒	☐	MRI-based neuroimaging

Antibodies

Antibodies used	Anti-SARS S2 (Abcam, ab272504); Anti-alpha-tubulin (Millipore MAB1864); Anti-MUC5AC (ThermoScientific 45M1); Anti-MUC5B

Antibodies used	[polyclonal rabbit against a MUC5B peptide (MAN5BII)]; Anti-CCSP (Sigma 07-623); Anti-phalloidin (Invitrogen A22287); Anti-tubulin (Millipore, AB_2210391), Anti-ACE2 (Invitrogen, SN0754)
Validation	We did not validated the commercially available antibodies in this study, as the manufacturers have validation data for them. For the antibodies for primary human cell immunofluorescent staining, these antibodies have been used in our previous publications.

Eukaryotic cell lines

Policy information about cell lines and Sex and Gender in Research

Cell line source(s)	Simian kidney cell line Vero-E6 (ATCC # CRL1586) and murine DBT cells were preserved in our group. A clonal furin-overexpressing Vero cell line was generated by using the Sleeping Beauty Transposon System as previously described by our group. A549-ACE2 stable cell line was generated by our group by using Sleeping Beauty transposon system. All the primary human airway cells were purchased from UNC Marsico Lung Institute cell bank, which are commercially available for other institutions as well. These primary cells were collected from lung transplant patients, and we are blinded from the donor demographic information. The A549-ACE2 cell line was generated by our lab by stably transducing the A549 cells with human ACE2 gene using lentivirus vector.
Authentication	We did not authenticate the cell lines.
Mycoplasma contamination	All cell lines were tested negative with mycoplasma.
Commonly misidentified lines (See ICLAC register)	No commonly misidentified cell line was used in this study.

Animals and other research organisms

Policy information about studies involving animals; ARRIVE guidelines recommended for reporting animal research, and Sex and Gender in Research

Laboratory animals	Syrian hamsters, BALB/c mice, hACE2-K18 mice. The age of these animal was mentioned in the method section. The animals were housed in facilities with 12h dark/light cycle, ambient temperature and appropriate humidity.
Wild animals	No wild animal was included in this study.
Reporting on sex	Syrian hamsters are all female; BALB/c mice are all female; hACE2-K18 mice were both male and female.
Field-collected samples	No field-collected sample was involved in this study.
Ethics oversight	All hamster studies were performed in accordance with University of Wisconsin-Madison IACUC protocol # V006426. All mouse studies were performed were performed in accordance with UNC Animal Care and Use Committee (IACUC) protocol # 20-074.

Note that full information on the approval of the study protocol must also be provided in the manuscript.
