# Supplementary figures and images for: Host range, transmissibility and antigenicity of a pangolin coronavirus
Source: Nat Microbiol. 2023 Sep 25;8(10):1820–33. doi: 10.1038/s41564-023-01476-x (PMC10522490; doi:10.1038/s41564-023-01476-x)

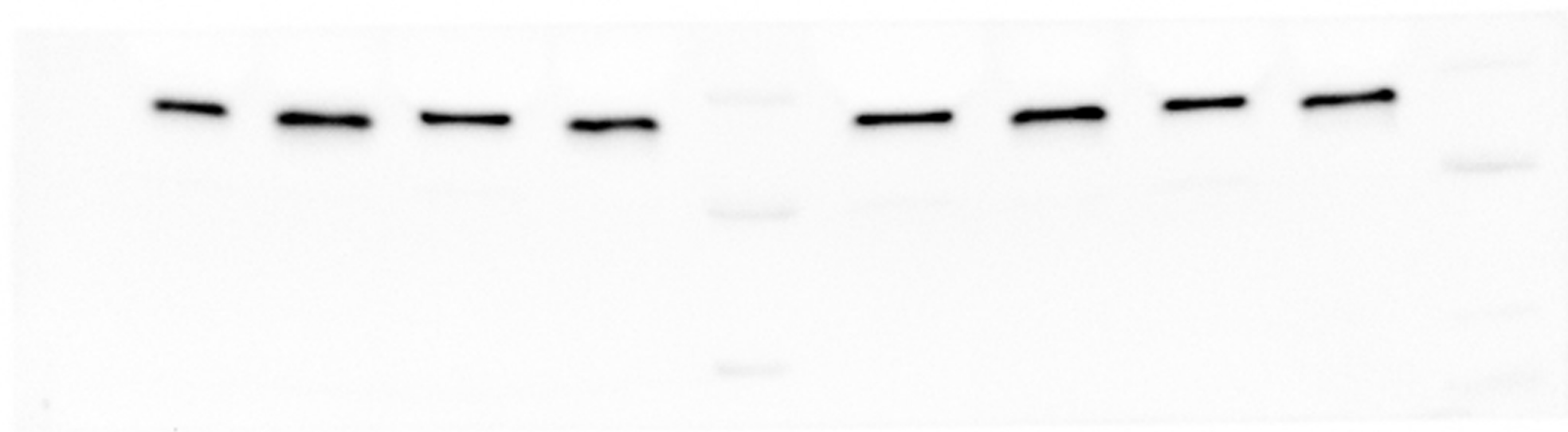

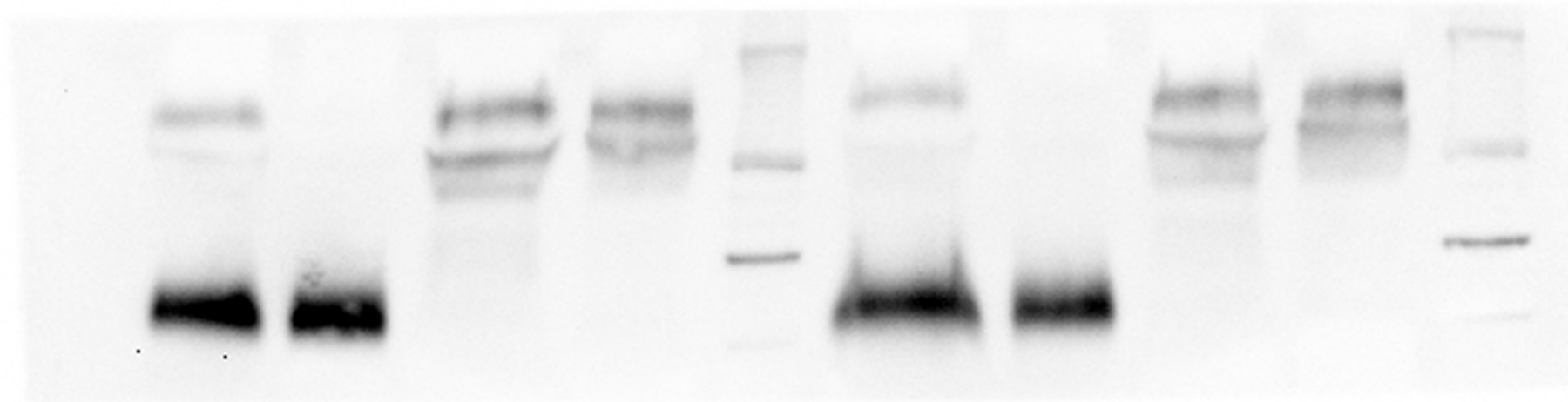

Supplement: Supplementary file 3 — Unprocessed western blot images for Fig. 1d. [file 41564_2023_1476_MOESM3_ESM.pdf]

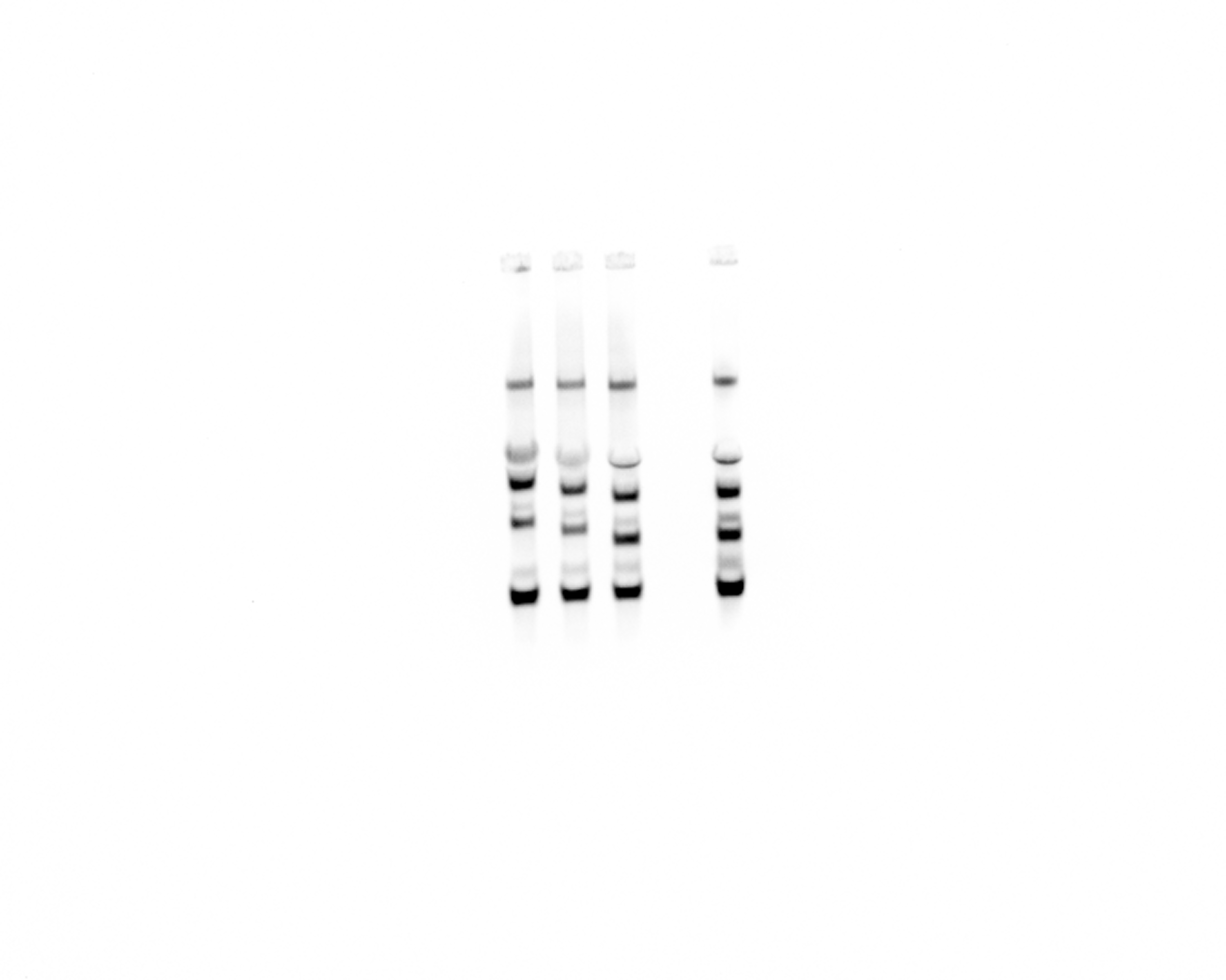

Supplement: Supplementary file 4 — Unprocessed northern blot images for Fig. 1c. [file 41564_2023_1476_MOESM4_ESM.tif]

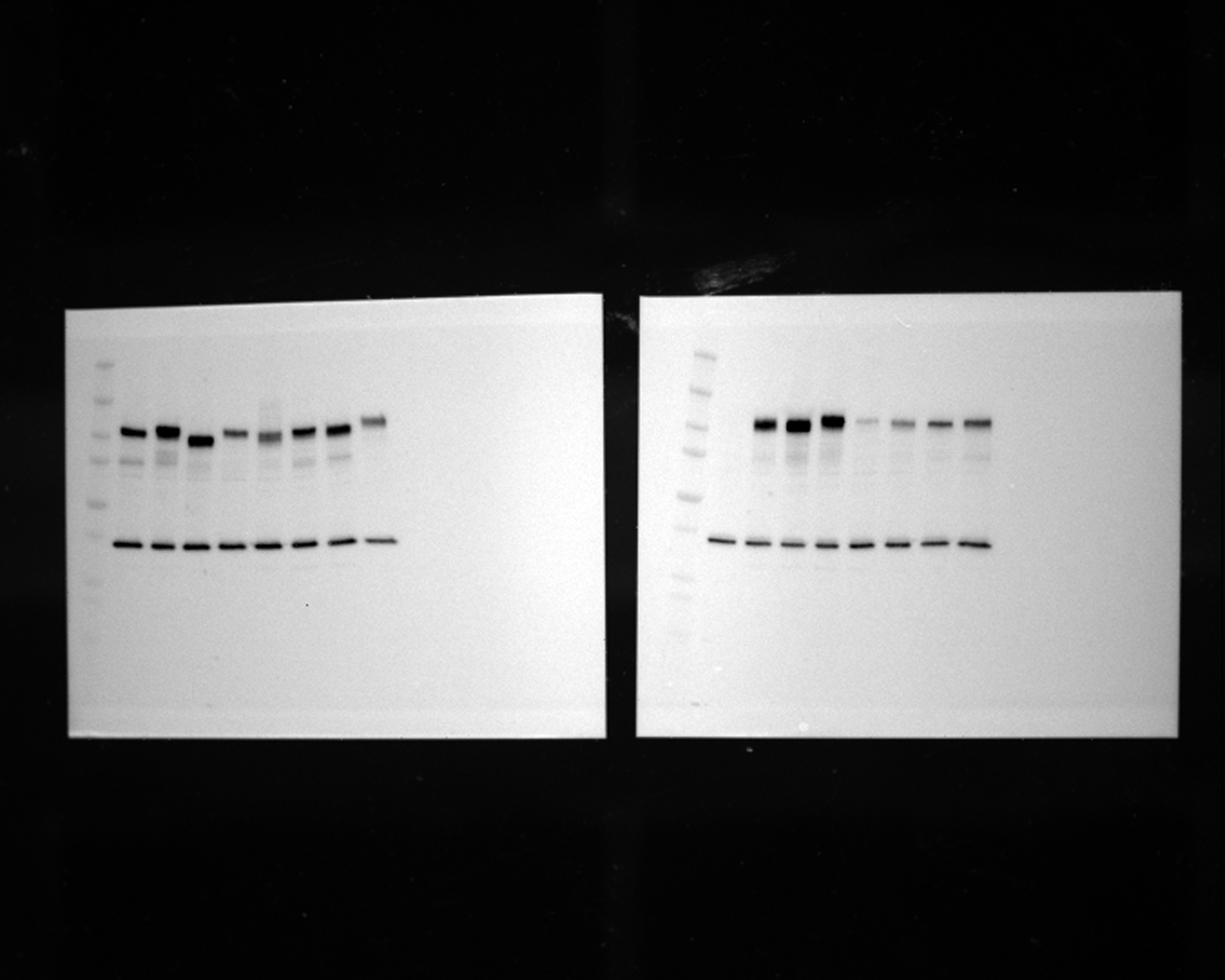

Supplement: Supplementary file 5 — Unprocessed western blot images for Fig. 2b. [file 41564_2023_1476_MOESM5_ESM.tif]
